# Survival at 3, 6 and 12 months in patients diagnosed with community-acquired pneumonia in Colombia: a retrospective cohort study

**DOI:** 10.1016/j.bjid.2024.103852

**Published:** 2024-07-20

**Authors:** Eduardo Tuta-Quintero, Daniela Torres-Arevalo, Alirio Rodrigo Bastidas-Goyes, Hermencia C. Aponte-Murcia, Manuela Guerrero, Andrea Giraldo, Laura Villarraga, Laura Orjuela, Juan Hernández, Luis F. Giraldo-Cadavid

**Affiliations:** a Universidad de La Sabana, School of Medicine, Chía, Colombia; b Clínica Universidad de La Sabana, Chía, Colombia; c Fundacion Neumologica Colombiana, Chief of the Interventional Pulmonology Service, Bogotá, Colombia

**Keywords:** Survival, Pneumonia, Risk factor, Observational study, Outcome

## Abstract

**Background:**

The primary aim of this study is to assess the survival rates of individuals diagnosed with Community-Acquired Pneumonia (CAP) post-hospitalization in Colombia. Additionally, explore potential risk factors associated with decreased long-term survival.

**Methods:**

A retrospective cohort study was conducted in a hospital in Colombia, evaluating survival at 3, 6 and 12 months in CAP patients, using the Kaplan–Meier method. Stratifications were made by age, sex, comorbidity, and severity. The comparison of survival curves was performed using the Log-Rank test, a multivariate analysis with Cox regression was performed to study possible risk factors that affected 12-month survival in patients with CAP.

**Results:**

3688 subjects were admitted, with a mortality of 16.3 % per year. Survival at three, six, and twelve months was 92.9 % (95 % CI 92–93 %), 88.8 % (95 % CI 87–90 %), and 84.2 % (95 % CI 82–85 %), respectively. Analysis stratified by pneumonia severity index, 12-month survival was 98.7 % in Class I, 95.6 % in Class II, 87.41 % in Class III, 77.1 % in Class IV, and 65.8 % in class-V (*p* < 0.001). Cox-regression showed that being male (HR = 1.44; 95 % CI 1.22‒1.70; *p* < 0.001), an elevated pneumonia severity index (HR = 4.22; 95 % CI 1.89‒9.43; *p* < 0.001), a high comorbidity index (HR = 2.29; 95 % CI 1.89‒2.84; *p* < 0.001) and vasopressor requirement (HR = 2.22; 95 % CI < 0.001) were associated with a lower survival at twelve months of follow-up.

**Conclusion:**

Survival in patients with CAP who require hospitalization decreases at 3, 6, and 12 months of follow-up, being lower in patients older than 65 years, men, high comorbidity, and in subjects with severe presentation of the disease.

## Introduction

Community-Acquired Pneumonia (CAP) is a respiratory disease that presents mortality rates that can reach up to 14 %.^1^, ^2^, ^3^ Despite advances in vaccination, early diagnosis, and antibiotic management, CAP continues to be responsible for 5–12 % of lower respiratory tract infections,^3^ with a considerable proportion of cases requiring hospitalization and admission to the Intensive Care Unit (ICU).^4^ The one-year mortality rate can reach up to 40 % in hospitalized patients, while in those who did not require hospitalization it is 29.1 %.^2^ These findings suggest that CAP may have short- and long-term health implications.^5^, ^6^, ^7^

Studies show a decrease in survival and an increase in mortality in patients hospitalized for CAP, compared to those who do not require hospitalization.^3^, ^4^, ^5^ However, there is limited scientific literature regarding the long-term survival of patients with CAP, especially in low-middle income countries, including Colombia. Therefore, the objective of this study is to determine the survival of patients diagnosed with CAP at 3, 6 and 12 months after their hospitalization in Colombia. In addition, to characterize the affected population, estimate survival data, evaluate survival according to comorbidities, severity, age and sex, and evaluate possible factors associated with decreased long-term survival.

## Methods

Retrospective cohort study in patients hospitalized at the Clínica de la Universidad de La Sabana in Chía, Colombia. Active follow-up was conducted in the registry, including patients diagnosed with CAP from January 1, 2010, to February 28, 2020. Data were collected retrospectively from fully anonymous computerized medical records between June 1, 2022 and December 31, 2023, with survival assessment for at least 12 months (until December 31, 2023).

### Selection criteria

The inclusion criteria that were considered for study entry were age over 18 years, hospitalization requirement for CAP diagnosis, and complete clinical history with paraclinical tests, chest X-Ray and chest computer tomography scan on admission. Patients who were admitted to hospital for palliative care measures, presented nosocomial superinfection during their hospital stay, ruled out the diagnosis of CAP during their hospitalization, and/or who died within 30 days after hospital admission were excluded.

CAP was defined as an acute illness associated with at least one of the following signs or symptoms: fever, new cough with or without sputum production, pleuritic chest pain, dyspnea, or altered breath sounds on auscultation.^2^^,^^8^ In addition to a chest X-Ray with the presence of alveolar or interstitial infiltrate, consolidation or cavitations with or without pleural effusion that appears within the first 48 h after hospitalization.^2^^,^^8^ This screening process was conducted by trained healthcare professionals, including physicians and specialized nursing staff, who assessed patients upon admission to the study center.

### Clinical variables

The variables described were age, sex, Charlson Comorbidity Index (CCI) and ever-smoking or currently smoking tobacco products based on self-reported, vital signs, state of consciousness, complete blood count, arterial oxygen pressure, arterial carbon dioxide pressure, bicarbonate, corrected bicarbonate, base excess, arterial oxygen saturation, lactate dehydrogenase, inspired fraction of oxygen, arterial oxygen pressure/inspired fraction of oxygen, creatinine, blood ureic nitrogen, chest X-Ray and chest computer tomographic, these data was obtained from medical records at the time of admission to the hospital. For the evaluation of the imaging results, the research team did not use any blinding method.

The ICU admission, use of invasive mechanical ventilation, use of corticosteroids, and diagnosis of septic shock, defined by the need for vasopressor to maintain a mean arterial pressure of 65 mmHg or greater and serum lactate level greater than 2 mmoL/L in the absence of hypovolemia^2^^,^^8^; these data was obtained from medical records at the time of discharge to the ICU. The dependent variable was survival evaluated at 3, 6 and 12 months from time zero corresponding to the diagnosis of CAP. The information regarding follow-up and survival was obtained through the review of death certificates (RUAF ‒ Unique Registration of Affiliates), which were simultaneously verified during the collection and review of clinical histories.

### Sample size

To calculate the sample size,^9^^,^^10^ we applied the Schoenfeld formula for survival analysis with an alpha of 0.05 % and 90 % power, assuming a proportion of 15.8 % in the exposed group, as reported in a previous study by Saldias et al.^9^ regarding the prevalence of significant comorbidities in our setting. The Hazard ratio of 0.66, derived from the association between comorbidities and mortality in the study by Saldías et al. indicated that a minimum of 2892 subjects in follow-up were required.

### Statistical analysis

The data was transcribed into the Research Electronic Data Capture (REDCap) software.^11^ To reduce the risk of data entry errors, at least two team researchers reviewed the information during the transcription process. Subjects were selected by simple random sampling from the list of patients seen during the study period. An imputation analysis of missing data was performed for variables with a loss of less than 10 %, applying weighted mean imputation for quantitative variables and logistic regression for qualitative variables.^12^ Variables with data loss greater than 10 % were excluded from the analysis. To ensure that imputation has not biased or altered the study results, a comparison was conducted between non-imputed and imputed results, confirming that there was no difference that significantly modified the original data.^12^ Quantitative variables were summarized by measures of central tendency and dispersion, using means and Standard Deviations (SD) for normal distributions, medians, and interquartile ranges, for non-normal distributions. The Anderson–Darling test was used to assess normality, considering a value of *p* < 0.05 as significant. The qualitative variables were summarized in frequencies and percentages.^12^ To compare quantitative variables, the Studentʼs *t* and Mann–Whitney *U* tests were used. according to the distribution of the data, and for the qualitative variables the Chi Square test was used.^12^

Survival at 3, 6 and 12 months was evaluated through tables, and it was plotted with the Kaplan–Meier method, and the Log-Rank test was used to evaluate the statistical differences in the survival curves, according to the independent variables.^12^ Time to event was used as the dependent variable in the Cox regression model with variables selected for inclusion by bivariate analysis, using a significance threshold of *p* < 0.2, and their biological plausibility in relation to death. Regression coefficients and corresponding p-values for each independent variable were calculated. Likewise, Hazard Ratios (HR) were determined for each variable, considering a significance level of *p* < 0.05 as a relevant statistical estimate.

Due to many potential variables that could confound or mediate the causal effect of the exposure on the outcome, a directed acyclic graph model was created to reduce bias and improve transparency and increase the precision of the analysis (Supplementary Fig. 1).

## Results

### Population characteristics

**Fig. 1:**
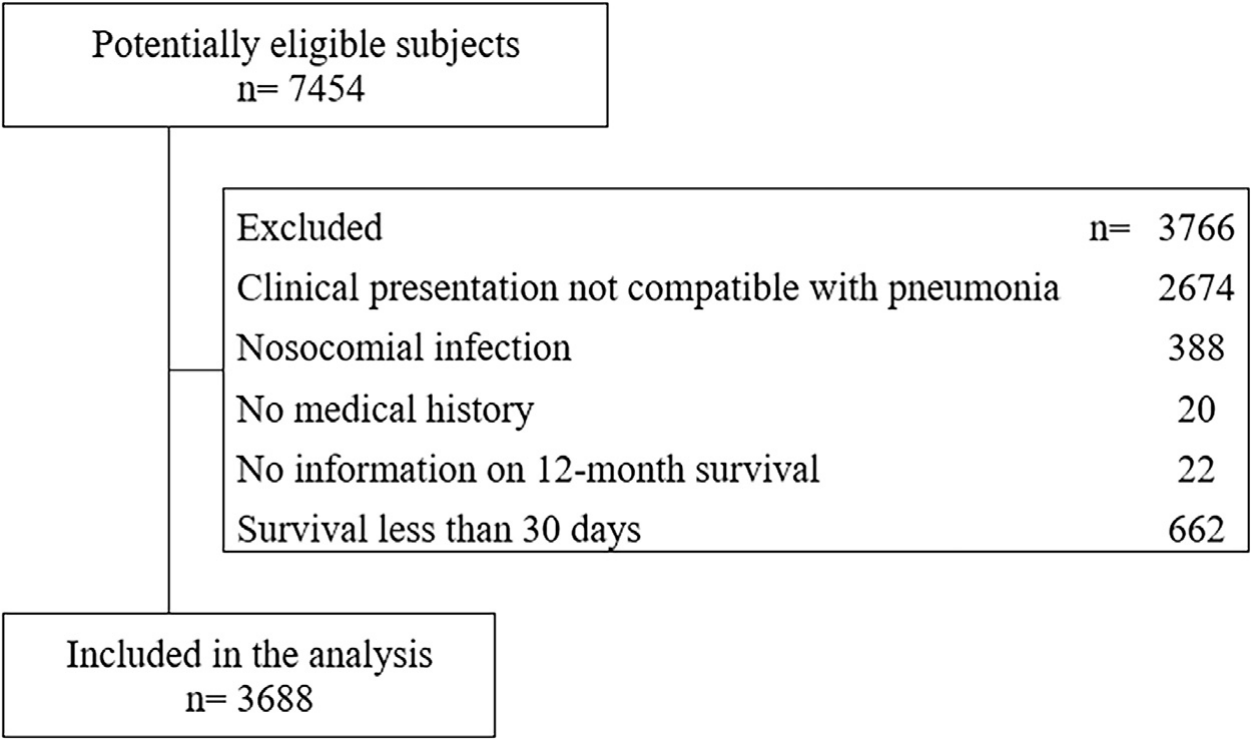
Flowchart of the admission of subjects to the study.

Of the 7454 patients initially included in the CAP cohort, 3688 patients were selected who met the established selection criteria (Fig. 1). The mean age was 63.5 years (SD = 21.39), and 2188 patients (59.3 %) were men (Table 1). The most frequent symptoms were cough (82.6 %), dyspnea (67.4 %), rales (52.2 %) and fever (47.6 %).

**Table 1:** General characteristics of the population.

	**Total population** **(*n*=3688)**	**Alive** **(*n*=3086)**	**Dead** **(*n*=602)**	**p-value**
Age in years, m (SD)	63.5 (21.39)	62 (21.78)	70.7 (17.53)	<0.001
Male gender, n (%)	2188 (59.3)	1801 (58.4)	387 (64.3)	0.007
Number of reconsultations, n (%)	3688 (0.78)	3086 (0.85)	602 (0.46)	<0.001
Cough, n (%)	3045 (82.6)	2586 (83.8)	459 (76.2)	<0.001
Dyspnoea, n (%)	2486 (67.4)	2095 (67.9)	391 (65)	0.160
Diarrhea, n (%)	220 (6)	186 (6)	34 (5.6)	0.718
Fever, n (%)	1756 (47.6)	1500 (48.6)	256 (42.5)	0.006
Pleuritic pain, n (%)	954 (25.9)	843 (27.3)	111 (18.4)	<0.001
Cyanosis, n (%)	234 (6.3)	190 (6.2)	44 (7.3)	0.290
Retractions, n (%)	731 (19.8)	605 (19.6)	126 (20.9)	0.458
Headache, n (%)	311 (8.4)	271 (8.8)	40 (6.6)	0.084
Altered consciousness, n (%)	371 (10.1)	261 (8.5)	110 (18.3)	<0.001
Rales, n (%)	1924 (52.2)	1628 (52.8)	296 (49.2)	0.106
Wheezing, n (%)	829 (22.5)	719 (23.3)	110 (18.3)	0.007
Lymph node pain, n (%)	19 (0.5)	15 (0.5)	4 (0.7)	0.576
Heart rate bpm, m (SD)	91.3 (18.45)	91.5 (18.3)	90.4 (19.22)	0.181
SBP mmHg, m (SD)	122.7 (21.49)	123.1 (20.69)	120.5 (25.25)	0.022
DBP mmHg, m (SD)	72.9 (13.14)	73.1 (12.73)	71.7 (15.13)	0.029
MAP mmHg, m (SD)	89.5 (14.6)	89.8 (14.01)	88 (17.31)	0.016
Respiratory rate bepm, m (SD)	21.3 (4.76)	21.2 (4.65)	22 (5.27)	<0.001
Temperature °C, m (SD)	36.9 (0.92)	36.9 (0.93)	36.8 (0.79)	<0.001
Oxygen saturation, n (%)	89.1 (6.7)	89.2 (6.57)	88.9 (7.33)	0.299
FiO_2_ upon admission, n (%)	28.5 (12.34)	28.1 (11.59)	30.3 (14.98)	<0.001

m, Average; SD, Standard Deviation; n, Number; bpm, Beats per minute; SBP, Systolic Blood Pressure; DBP, Diastolic Blood Pressure; MAP, Mean Arterial Pressure; bepm, Breaths per minute; FiO_2_, Inspired fraction of Oxygen.

### History, laboratory, and chest diagnostic images

Dead patients had higher rates of hypertension (52.1 % vs. 44.9 %, *p* = 0.001), chronic heart failure (15 % vs. 11.6 %, *p* = 0.020), acute myocardial infarction (5.3 % vs. 4.4 %, *p* < 0.001), and cerebrovascular disease (10.8 % vs. 6.3 %, *p* < 0.001) (Supplementary Table 1). dead patients had lower hemoglobin (13 vs. 13.7 g/dL, *p* < 0.001) and hematocrit (38.8% vs. 40.7 %, *p* < 0.001) (Supplementary Table 2). In chest X-Rays, dead patients had a higher incidence of interstitial infiltrates (55.5 % vs. 46.4 %, *p* < 0.001), atelectasis (10.6 % vs. 7.3 %, *p* = 0.007), bilateral consolidation (26.5 % vs. 13.8 %, *p* < 0.001), and presence of pneumonia affecting multiple lobes of the lung simultaneously (32.9 % vs. 18 %, *p* < 0.001) (Supplementary Table 3).

### Medical treatment

12.1 % of the patients who died required management with vasopressor support, in contrast to 6.4 % of those who survived (*p* < 0.001). 17.6 % of the patients who died required management in the ICU (Table 2).

**Table 2:** Medical treatment.

	**Total population** **(*n*=3688)**	**Alive** **(*n*=3086)**	**Dead** **(*n*=602)**	**p-value**
Septic shock, n (%)	271 (7.3)	198 (6.4)	73 (12.1)	<0.001
Vasopressor support, n (%)	260 (7.3)	182 (6.4)	78 (12.1)	<0.001
Use of corticosteroids, n (%)	767 (20.8)	577 (18.7)	190 (31.6)	<0.001
Hydrocortisone, n (%)	252 (32.9)	201 (34.9)	51 (26.8)	0.040
Methylprednisolone, n (%)	233 (30.4)	170 (29.5)	63 (33.2)	0.344
Prednisone, n (%)	257 (33.5)	202 (35)	55 (28.9)	0.125
Intensive Care Unit, n (%)	414 (11.2)	308 (10)	106 (17.6)	<0.001
Intensive Care Unit stay days, m (SD)	11.6 (20.25)	9.6 (11.93)	17.5 (34.47)	<0.001
Invasive Mechanical Ventilation, n (%)	266 (7.2)	200 (6.5)	66 (11)	<0.001
Non-Invasive Mechanical Ventilation, n (%)	132 (3.6)	94 (3)	38 (6.3)	<0.001
Hospitalization requirement, n (%)	3172 (86)	2621 (84.9)	551 (91.5)	<0.001
Length of stay days, m (SD)	10.7 (83.25)	10.3 (90.71)	12.7 (16.19)	0.180

n, Number; m, Average; SD, Standard Deviation.

### Survival analysis

**Fig. 2:**
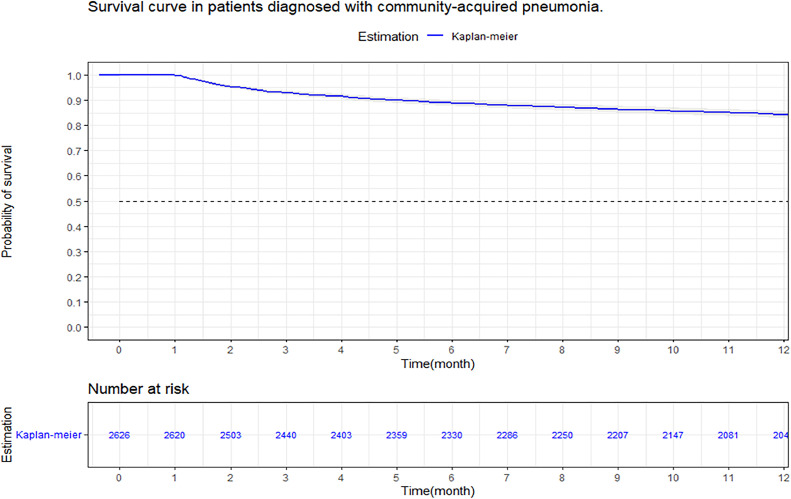
Kaplan–Meier survival curves.

**Fig. 3:**
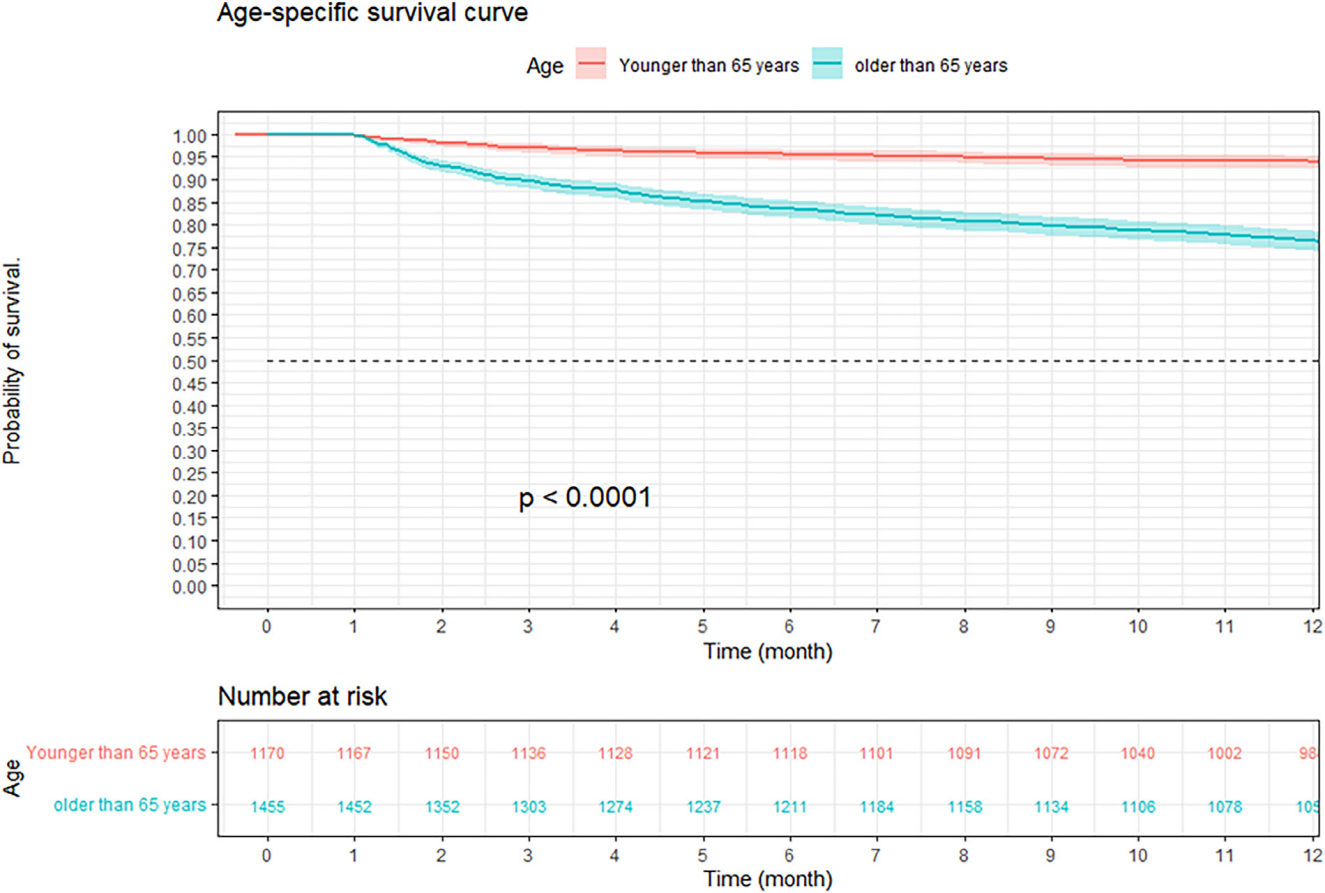
Kapla–Meier survival curves of hospitalized adult patients with community-acquired pneumonia according to age.

**Fig. 4:**
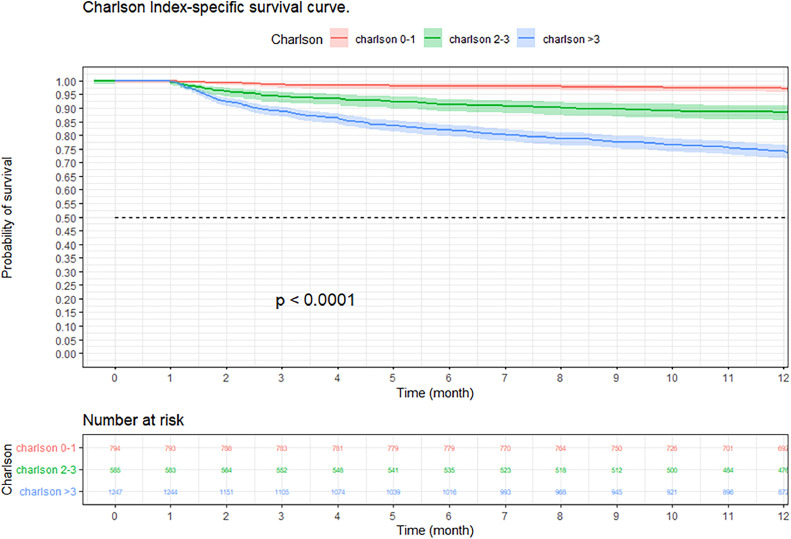
Kaplan–Meier survival curves of hospitalized adult patients with community-acquired pneumonia according to comorbidity. Notes: The p-value represents the statistically significant difference between patients with significant comorbidities and patients with mild comorbidities in survival at 3, 6, and 12 months.

**Fig. 5:**
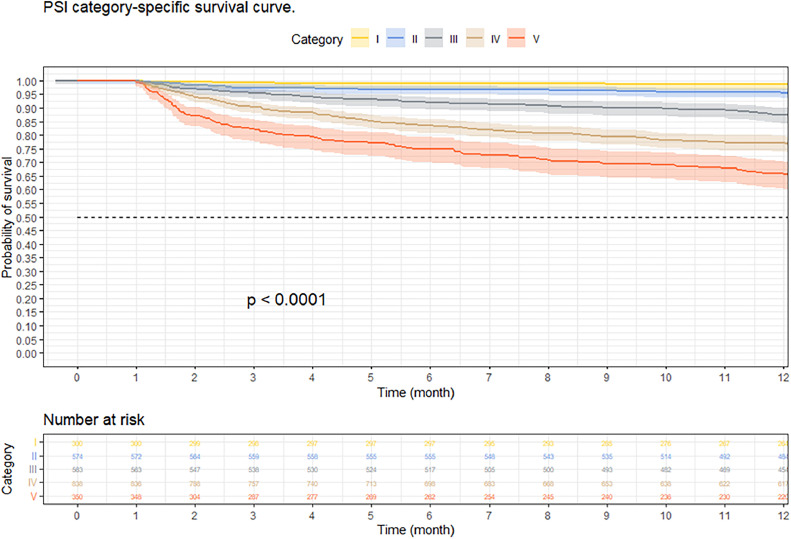
Kaplan–Meier survival curves of hospitalized adult patients with community-acquired pneumonia according to pneumonia severity index.

At three months, the survival rate was 92.9 % (95 % CI 92–93 %), at six months it was 88.8 % (95 % CI 87–90 %), and at 12-months it was 84.2 % (95 % CI 82–85 %) (Fig. 2). When performing an analysis stratified by age (> 65 years and < 65 years), a survival of 89.6 %, 83.4 %, and 76.4 % was found in the group older than 65 years, in contrast to 97.1 %, 95.5 %, and 94 % in the group younger than 65 years (*p* < 0.001) (Fig. 3). Regarding sex, survival was 91.9 %, 88.1 %, and 83.2 % in men, while in women it was 94.3 %, 89.8 %, and 85.7 % (*p* = 0.05) (Supplementary Fig. 2). The presence with significant comorbidities (CCI ≥ 3) was associated with a survival of 88.7 %, 88.8 %, and 74.1 %, compared with those with mild comorbidities (CCI 0‒1), who had a survival of 98.6 %, 98.1 %, and 97.2 % (*p* < 0.001) (Fig. 4). In relation to severity according to the Pneumonia Severity Index (PSI), survival at 12 months was 98.7 % in Class I, 95.6 % in Class II, 87.41 % in Class III, 77.1 % in Class IV, and 65.8 % in Class V (*p* < 0.001) (Fig. 5). The Nelson-Aalen risk function estimation curve is shown in (Supplementary Fig. 3).

The results of the age stratification showed that older patients (≥ 65-years) had lower 12-month survival than younger patients (< 65-years), regardless of the CCI. In older patients, 12-month survival was 80 %, 85.9 %, and 74 %, respectively, for CCIs of 0‒1, 2‒3, and > 3. In younger patients, 12-month survival was 97.3 %, 91.2 %, and 74.8 %, respectively, for the same CCI (Supplementary Figs. 4‒5).

### Cox regression: independently associated characteristics with long-term mortality

Cox regression showed that being male (HR = 1.44; 95 % CI 1.22‒1.70; 0 < 0.001), an elevated PSI (HR = 4.22; 95 % CI 1.89‒9.43; *p* < 0.001), a high comorbidity index (HR = 2.29; 95 % CI 1.89‒2.84; *p* < 0.001) and vasopressor requirement (HR = 2.22; 95 % CI < 0.001) were associated with a lower survival at twelve months of follow-up (Table 3).

**Table 3:** Cox regression: independently associated characteristics with long-term mortality.

**Variable**	**HR**	**95 % CI**	**p-value**
Male gender	1.44	1.22‒1.70	<0.001
Age over 65 years	1.35	1.01‒1.81	0.040
PSI Score	1.007	1.004‒1.01	
Category II	1.75	0.81‒3.78	0.14
Category III	2.43	1.11‒5.31	0.025
Category IV	3.03	1.37‒6.68	0.005
Category V	4.22	1.89‒9.43	<0.001
Charlson Comorbidity Index	2.29	1.84‒2.84	<0.001
Multilobar involvement^a^	1.24	1.04‒1.48	0.013
Vasopressor support requirement ‒ time interaction	2.22	1.68‒2.93	<0.001
		Concordance: 74%	
		p-value < 0.001	

PSI, Pneumonia Severity Index; HR, Hazard Ratio.

a Presence of pneumonia affecting multiple lobes of the lung simultaneously.

## Discussion

This study evaluated survival at 3, 6, and 12 months in patients hospitalized for CAP at a university hospital in Colombia. Clinical factors associated with decreased survival were identified. The main findings include a survival rate of 92.9 % at 3 months, 88.8 % at 6 months, and 84.2 % at 12 months. A lower survival was observed in patients older than 65 years, with more comorbidities and a greater severity of pneumonia. The most common comorbidities were arterial hypertension, chronic obstructive pulmonary disease, and diabetes mellitus, more frequently in deceased patients. In addition, paraclinical findings were found in the deceased patients, such as increased renal compromise, requirement of vasopressor support, and admission to the ICU. Cox regression showed a significant association between the decrease in one-year survival and the variables of sex, age, CCI and PSI, vasopressor support requirement, and presence of pneumonia affecting multiple lobes of the lung simultaneously.

Age is an influential variable in the decrease in one-year survival in patients diagnosed with CAP.^13^ This relationship can be attributed to the higher prevalence of comorbidities, a weakened immune response, and increased exposure to various pathogens in elderly individuals.^14^ In a study conducted by Johnstone et al.^15^ in 2008 in a Canadian hospital, long-term survival was analyzed in patients hospitalized for CAP. During a one-year follow-up, a reduction in survival was observed in those patients older than 65 years, compared to the younger ones. Likewise, other studies support the association between advanced age and higher long-term mortality in patients with CAP. For example, the work of Saldías et al.^9^ who evaluated a cohort of patients in Chile, and found results consistent with those of this study.

Comorbidities also play a significant role in decreasing long-term survival in patients with CAP.^16^, ^17^, ^18^ These concomitant medical conditions can alter the hostʼs inflammatory and immunological response, promote bacterial colonization of the respiratory tract, and increase the risk of complications.^17^ In this study, the presence of comorbidities was evaluated using the CCI, following the approach of other studies carried out in different countries. The findings indicate that the CCI is an independent predictor of long-term mortality (HR = 2.29; 95 % CI 1.84‒2.84). These results are consistent with the previous findings of Bordon et al. in a study conducted in Kentucky in 2010.^6^ Furthermore, the study conducted by Tokgoz et al.^19^ at a hospital in Turkey also found the CCI to be an independent predictor of long-term mortality with an HR of 1.180 (< 0.0001), which supports their findings.

The severity of CAP was assessed using PSI,^20^ an instrument widely used in survival studies to examine its association with its decrease. In line with the results reported in previous studies, such as that of Cecere et al.^21^ in which a higher PSI category is associated with decreased long-term survival, the findings of our study also indicate that PSI categories IV and V are associated with significantly decreased survival, compared with the lowest categories, such as I or II. These results are supported by a study conducted by Ruiz et al.^22^ in Spain, where a survival of 47.6 % was found in category V of the PSI; a figure lower than that found in this study (65.8 %), but equally significant.

It was observed that the presence of elevated blood urea nitrogen levels, leukopenia, and presence of pneumonia affecting multiple lobes of the lung simultaneously were more frequent in those patients who died at the end of follow-up. These findings are consistent with what was described by Welte et al.^23^ in a narrative review, in which he mentions that elevated blood urea nitrogen, low leukocyte count, and the presence of pneumonia affecting multiple lobes of the lung simultaneously are predictors of mortality and severity in patients with CAP. Furthermore, our results agree with the findings reported by Yoshimoto et al. who demonstrated that these factors were associated with an increased risk of mortality in patients with CAP.^24^ Taken together, these studies support the importance of identifying and monitoring these risk factors as an integral part of the clinical approach to CAP.^19^^,^^25^ Additionally, the study by Surme et al.^26^ also found that the presence of hypotension (systolic blood pressure < 90 mmHg) and the need for invasive mechanical ventilation were independent predictors of long-term mortality in patients with CAP.

Among the limitations of the study, the exclusive inclusion of patients from a single hospital stands out, which limits the generalization of the results to other populations. On the other hand, there is a selection bias due to the exclusion of patients who died within 30 days, patients who developed nosocomial infections, and patients with incomplete data, which is another very important limitation that compromises the veracity of the survival estimates. Although measures were taken to minimize selection bias, it is important to consider that the results may be different in patients from other hospitals or in patients from different countries. Despite being a retrospective study based on medical records, measures were implemented to minimize information bias, such as the training of the personnel in charge of collecting medical data and the construction of the manuscript based on the STROBE checklist of items that should be included in cohort study reports (Supplementary Table 4).

The lack of updating and incomplete data in the information sources limited the analysis of the immunization variable. At least 6 to 10 of the statistically significant findings in this study are false positives due to the large number of hypothesis tests performed in the analysis of our results. Furthermore, collinearity between predictor variables can inflate regression coefficients and limit the interpretation of results. These findings highlight the importance of future studies to evaluate the impact of chronic disease control after hospitalization in patients with CAP, including measures such as early control of renal failure and optimization of vasopressor support, with the aim of improving both the short-term and long-term prognosis.

## Conclusion

Survival in patients with CAP who require hospitalization decreases at 3, 6, and 12 months of follow-up, being lower in patients older than 65 years, men, with high comorbidity, and in subjects with severe presentation of the disease. These findings highlight the importance of prevention strategies, early diagnosis, and timely treatment of CAP, especially in high-risk patients.

## Ethical approval and consent to participate

This study was conducted in compliance with the Declaration of Helsinki. The study was approved by Ethics Committee in Academic Research of the Clínica Universidad de La Sabana (Code: 20220102). All methods were performed in accordance with the relevant guidelines and regulations. Consent was waived given the retrospective nature of this study.

## Consent for publication

Not Applicable.

## Availability of data and materials

The datasets utilized and analyzed in the present study are accessible from the corresponding author upon reasonable request.

## Authors’ contributions

Conceptualization, data curation and formal analysis were performed by ETQ, DTA, ABG, and LFGC. Investigation, software analysis and laboratory assays were performed by ETQ, DTA, ABG, and LFGC. Writing, editing, and review were performed by ETQ, DTA, ABG, HCA, MG, AG, LV, LO and JH. All authors read and approved the final manuscript.

## Funding

This work was supported by Universidad de la Sabana Grant MEDMSc-69-2023.

## Conflicts of interest

The authors declare no conflicts of interest.

## Appendix Supplementary materials

Image, application 1
