# Supplementary material for: Survival at 3, 6 and 12 months in patients diagnosed with community-acquired pneumonia in Colombia: a retrospective cohort study
Source: Braz J Infect Dis. 2024 Jul 20;28(4):103852. doi: 10.1016/j.bjid.2024.103852 (PMC11327535; doi:10.1016/j.bjid.2024.103852)

**BJID-D-24-00080_ Supplementary Material**

**Supplementary Table 1** Comorbidities.

|  | **Total population**  **(n = 3688)** | **Alive**  **(n = 3086)** | **Dead**  **(n = 602)** | **p-value** |
| --- | --- | --- | --- | --- |
| Hypertension, n (%) | 1699 (46.1) | 1386 (44.9) | 313 (52.1) | 0.001 |
| Smoking, n (%) | 668 (18.1) | 547 (17.7) | 121 (20.1) | 0.163 |
| Chronic heart failure, n (%) | 447 (12.1) | 357 (11.6) | 90 (15) | 0.020 |
| Acute myocardial infarction, n (%) | 168 (4.6) | 136 (4.4) | 32 (5.3) | <0.001 |
| Peripheral vascular disease, n (%) | 110 (3) | 88 (2.9) | 22 (3.7) | 0.287 |
| Cerebrovascular disease, n (%) | 484 (7) | 251 (6.3) | 233 (10.8) | <0.001 |
| Chronic lung disease, n (%) | 941 (25.5) | 770 (25) | 171 (28.5) | 0.073 |
| Ulcer disease, n (%) | 48 (1.3) | 36 (1.2) | 12 (2) | 0.101 |
| Liver disease, n (%) | 22 (0.6) | 16 (0.5) | 6 (1) | 0.163 |
| Moderate-severe liver disease, n (%) | 14 (0.4) | 10 (0.3) | 4 (0.7) | 0.214 |
| Diabetes Mellitus without end-organ damage, n (%) | 423 (11.5) | 342 (11.1) | 81 (13.5) | 0.093 |
| Diabetes Mellitus with end-organ damage, n (%) | 104 (2.8) | 83 (2.7) | 21 (3.5) | 0.277 |
| Mild chronic kidney disease, n (%) | 150 (4.1) | 111 (3.6) | 39 (6.5) | 0.001 |
| Moderate-severe chronic kidney disease, n (%) | 201 (5.5) | 149 (4.8) | 52 (8.7) | <0.001 |
| Tumor, n (%) | 237 (6.4) | 168 (5.4) | 69 (11.5) | <0.001 |
| Metastasis, n (%) | 60 (1.6) | 37 (1.2) | 23 (3.8) | <0.001 |
| Leukemia, n (%) | 26 (0.7) | 14 (0.5) | 12 (2) | <0.001 |
| Lymphoma, n (%) | 24 (0.7) | 19 (0.6) | 5 (0.8) | 0.547 |
| HIV/AIDS, n (%) | 18 (0.5) | 13 (0.4) | 5 (0.8) | 0.187 |
| Connective tissue disease, n (%) | 110 (3) | 90 (2.9) | 20 (3.3) | 0.589 |
| Dementia, n (%) | 284 (7.7) | 201 (6.5) | 83 (13.8) | <0.001 |
| Hemiplegia, n (%) | 108 (2.9) | 82 (2.7) | 26 (4.3) | 0.027 |
| Asthma, n (%) | 79 (2.1) | 74 (2.4) | 5 (0.8) | 0.015 |
| Immunosuppression, n (%) | 148 (4) | 115 (3.7) | 33 (5.5) | 0.043 |
| Trauma, n (%) | 88 (2.4) | 70 (2.3) | 18 (3) | 0.284 |
| Functional status, n (%) | 494 (13.4) | 359 (11.6) | 135 (22.4) | <0.001 |
| Institutionalized, n (%) | 445 (12.1) | 350 (11.3) | 95 (15.8) | 0.002 |

n, Number; HIV/AIDS, Human Immunodeficiency Virus/Acquired Immunodeficiency Syndrome.

**Supplementary Table 2** Arterial blood gases and laboratory tests.

|  | **Total population**  **(n = 3688)** | **Alive**  **(n = 3086)** | **Dead**  **(n = 602)** | **p-value** |
| --- | --- | --- | --- | --- |
| pH, m (SD) | 7.42 (0.06) | 7.42 (0.06) | 7.43 (0.07) | 0.002 |
| PaO_2_, m (SD) | 62.1 (19.32) | 61.8 (18.09) | 63.3 (23.79) | 0.138 |
| PaCO_2_, m (SD) | 33 (8.32) | 33 (8.29) | 32.7 (8.45) | 0.329 |
| HCO_3_, m (SD) | 21 (4.02) | 20.9 (3.9) | 21.4 (4.45) | 0.009 |
| C-HCO_3_, m (SD) | 22.3 (3.49) | 22.2 (3.18) | 22.6 (4.04) | 0.021 |
| Base excess, m (SD) | -2.5 (3.92) | -2.6 (3.8) | -2.1 (4.32) | 0.004 |
| LDH, m (SD) | 2 (1.32) | 2 (1.28) | 1.9 (1.43) | 0.013 |
| SO_2_, m (SD) | 88.7 (7.79) | 88.7 (7.83) | 88.8 (7.63) | 0.823 |
| FiO_2_, m (SD) | 28.5 (12.34) | 28.1 (11.59) | 30.3 (14.98) | <0.001 |
| PaO_2_/FiO_2_, m (SD) | 233 (69.7) | 233.8 (69) | 229.5 (72.55) | 0.163 |
| Leukocytes cells ×10^3, m (SD) | 12281.4 (6204.46) | 12410.7 (6235.79) | 11632.8 (6008.02) | 0.005 |
| Hemoglobin g/dL, m (SD) | 13.5 (2.39) | 13.7 (2.27) | 13 (2.83) | <0.001 |
| Hematocrit %, m (SD) | 40.4 (6.78) | 40.7 (6.55) | 38.8 (7.66) | <0.001 |
| Platelets cells ×10^3, m (SD) | 259.6 (102.24) | 259.4 (100.93) | 260.7 (108.52) | 0.779 |
| Sodium meq/L, m (SD) | 137.2 (5.86) | 137 (5.44) | 138.1 (7.32) | <0.001 |
| Glucose mg/dL, m (SD) | 134.1 (67.66) | 133.9 (67.25) | 135.2 (69.48) | 0.654 |
| Albumin g/dL, m (SD) | 3 (1.29) | 3 (0.86) | 3 (2.12) | 0.680 |
| Creatinine mg/dL, m (SD) | 1.3 (3.2) | 1.3 (3.46) | 1.4 (1.45) | 0.370 |
| BUN mg/dL, m (SD) | 23.3 (16.83) | 22.5 (16.25) | 27.2 (18.83) | <0.001 |

m, average; SD, Standard Deviation; n, number; PaO_2_, Arterial Oxygen pressure; PaCO_2_, Arterial Carbon Dioxide pressure; HCO_3_, Bicarbonate; C-HCO_3_, Corrected Bicarbonate; BE, Base Excess; SaO_2_, Arterial Oxygen Saturation; LDH, Lactate Dehydrogenase; FiO_2_, Inspired Fraction of Oxygen; PaO_2_/FiO_2_ ratio, Arterial Oxygen Pressure/Inspired Fraction of Oxygen; BUN, Blood Ureic Nitrogen.

**Supplementary Table 3** Chest X-Ray and Chest computed tomography scan.

|  | **Total population**  **(n = 3688)** | **Alive**  **(n = 3086)** | **Dead**  **(n = 602)** | **p-value** |
| --- | --- | --- | --- | --- |
| **Chest X-Ray, n (%)** |  |  |  |  |
| Interstitial Infiltrate | 1744 (47.8) | 1415 (46.4) | 329 (55.5) | <0.001 |
| Alveolar Infiltrates | 2351 (64.5) | 1989 (65.2) | 362 (61) | 0.055 |
| Atelectasis | 287 (7.9) | 224 (7.3) | 63 (10.6) | 0.007 |
| Unilateral Atelectasis | 241 (84) | 188 (83.9) | 53 (84.1) | 0.970 |
| Bilateral Atelectasis | 45 (15.7) | 35 (15.6) | 10 (15.9) | 0.962 |
| Consolidation | 2263 (62.1) | 1919 (62.9) | 344 (58) | 0.025 |
| Unilateral Consolidation | 1890 (83.5) | 1636 (85.3) | 254 (73.8) | <0.001 |
| Bilateral Consolidation | 355 (15.7) | 264 (13.8) | 91 (26.5) | <0.001 |
| Multilobar | 745 (20.4) | 550 (18) | 195 (32.9) | <0.001 |
| Effusion | 495 (13.6) | 417 (13.7) | 78 (13.2) | 0.740 |
| Unilateral Effusion | 346 (69.9) | 290 (69.5) | 56 (71.8) | 0.691 |
| Bilateral Effusion | 143 (28.9) | 121 (29) | 22 (28.2) | 0.885 |
| **Chest CT scan, n (%)** |  |  |  |  |
| Interstitial Infiltrate | 533 (45.8) | 386 (43.7) | 147 (52.5) | 0.010 |
| Alveolar Infiltrates | 786 (100) | 618 (78.6) | 168 (21.4) | 0.002 |
| Atelectasis | 188 (16.2) | 148 (16.8) | 40 (14.3) | 0.327 |
| Unilateral Atelectasis | 136 (72.3) | 108 (73) | 28 (70) | 0.709 |
| Bilateral Atelectasis | 52 (27.7) | 39 (26.4) | 13 (32.5) | 0.441 |
| Consolidation | 844 (72.6) | 651 (73.7) | 193 (69.2) | 0.137 |
| Unilateral Consolidation | 525 (62.2) | 435 (66.8) | 90 (46.6) | <0.001 |
| Bilateral Consolidation | 315 (37.4) | 211 (32.5) | 104 (53.9) | <0.001 |
| Multilobar | 526 (45.3) | 373 (42.2) | 153 (54.8) | <0.001 |
| Effusion | 314 (27) | 242 (27.4) | 72 (25.8) | 0.600 |
| Unilateral Effusion | 205 (65.3) | 166 (68.6) | 39 (54.2) | 0.024 |
| Bilateral Effusion | 109 (34.7) | 76 (31.4) | 33 (45.8) | 0.024 |

n, Number; CT, Computed Tomography.

**Supplementary Table 4** STROBE Statement-Checklist of items that should be included in reports of cohort studies.

|  | **Item Nº** | **Recommendation** | **Page Nº** |
| --- | --- | --- | --- |
| **Title and abstract** |  | a) Indicate the study’s design with a commonly used term in the title or the abstract. |  |
|  |  | b) Provide in the abstract an informative and balanced summary of what was done and what was found |  |
| **Introduction** |  |  |  |
| Background/rationale | 2 | Explain the scientific background and rationale for the investigation being reported | 2‒3 |
| Objectives | 3 | State specific objectives, including any prespecified hypotheses | 2‒3 |
| **Methods** |  |  |  |
| Study design | 4 | Present key elements of study design early in the paper | 3 |
| Setting | 5 | Describe the setting, locations, and relevant dates, including periods of recruitment, exposure, follow-up, and data collection | 3 |
| Participants | 6 | a) Give the eligibility criteria, and the sources and methods of selection of participants. Describe methods of follow-up | 3 |
|  |  | b) For matched studies, give matching criteria and number of exposed and unexposed |  |
| Variables | 7 | Clearly define all outcomes, exposures, predictors, potential confounders, and effect modifiers. Give diagnostic criteria, if applicable | 4 |
| Data sources/ measurement | 8^a^ | For each variable of interest, give sources of data and details of methods of assessment (measurement). Describe comparability of assessment methods if there is more than one group | 4 |
| Bias | 9 | Describe any efforts to address potential sources of bias | 4‒5 |
| Study size | 10 | Explain how the study size was arrived at | 4‒5 |
| Quantitative variables | 11 | Explain how quantitative variables were handled in the analyses. If applicable, describe which groupings were chosen and why | 4‒5 |
| Statistical methods | 12 | a) Describe all statistical methods, including those used to control for confounding | 4‒5 |
|  |  | b) Describe any methods used to examine subgroups and interactions |  |
|  |  | c) Explain how missing data were addressed |  |
|  |  | d) If applicable, explain how loss to follow-up was addressed |  |
|  |  | e) Describe any sensitivity analyses |  |
| **Results** |  |  |  |
| Participants | 13^a^ | a) Report numbers of individuals at each stage of study ‒ e.g., numbers potentially eligible, examined for eligibility, confirmed eligible, included in the study, completing follow-up, and analyzed | 5 |
|  |  | b) Give reasons for non-participation at each stage |  |
|  |  | c) Consider use of a flow diagram |  |
| Descriptive data | 14^a^ | a) Give characteristics of study participants (e.g., demographic, clinical, social) and information on exposures and potential confounders | 5‒6 |
|  |  | b) Indicate number of participants with missing data for each variable of interest |  |
|  |  | c) Summarize follow-up time (e.g., average and total amount) |  |
| Outcome data | 15^a^ | Report numbers of outcome events or summary measures over time | 6 |
| Main results | 16 | a) Give unadjusted estimates and, if applicable, confounder-adjusted estimates and their precision (e.g., 95% Confidence Interval). Make clear which confounders were adjusted for and why they were included | 5‒6 |
|  |  | b) Report category boundaries when continuous variables were categorized |  |
|  |  | c) If relevant, consider translating estimates of relative risk into absolute risk for a meaningful time period |  |
| Other analyses | 17 | Report other analyses done ‒ e.g., analyses of subgroups and interactions, and sensitivity analyses | 6 |
| **Discussion** |  |  |  |
| Key results | 18 | Summarise key results with reference to study objectives |  |
| Limitations | 19 | Discuss limitations of the study, taking into account sources of potential bias or imprecision. Discuss both direction and magnitude of any potential bias | 7 |
| Interpretation | 20 | Give a cautious overall interpretation of results considering objectives, limitations, multiplicity of analyses, results from similar studies, and other relevant evidence | 7 |
| Generalisability | 21 | Discuss the generalizability (external validity) of the study results | 7 |
| **Other information** |  |  |  |
| Funding | 22 | Give the source of funding and the role of the funders for the present study and, if applicable, for the original study on which the present article is based | 7‒8 |

^a^ Give information separately for exposed and unexposed groups.

An Explanation and Elaboration article discusses each checklist item and gives methodological background and published examples of transparent reporting. The STROBE checklist is best used in conjunction with this article (freely available on the Web sites of PLoS Medicine at http://www.plosmedicine.org/, Annals of Internal Medicine at http://www.annals.org/, and Epidemiology at http://www.epidem.com/). Information on the STROBE Initiative is available at http://www.strobe-statement.org.

Supplementary Figure 1 Directed acyclic graph model of 12-month survival in patients with community-acquired pneumonia. (A) Directed acyclic graph model; (B) Representation of the variables using DAGitty software.


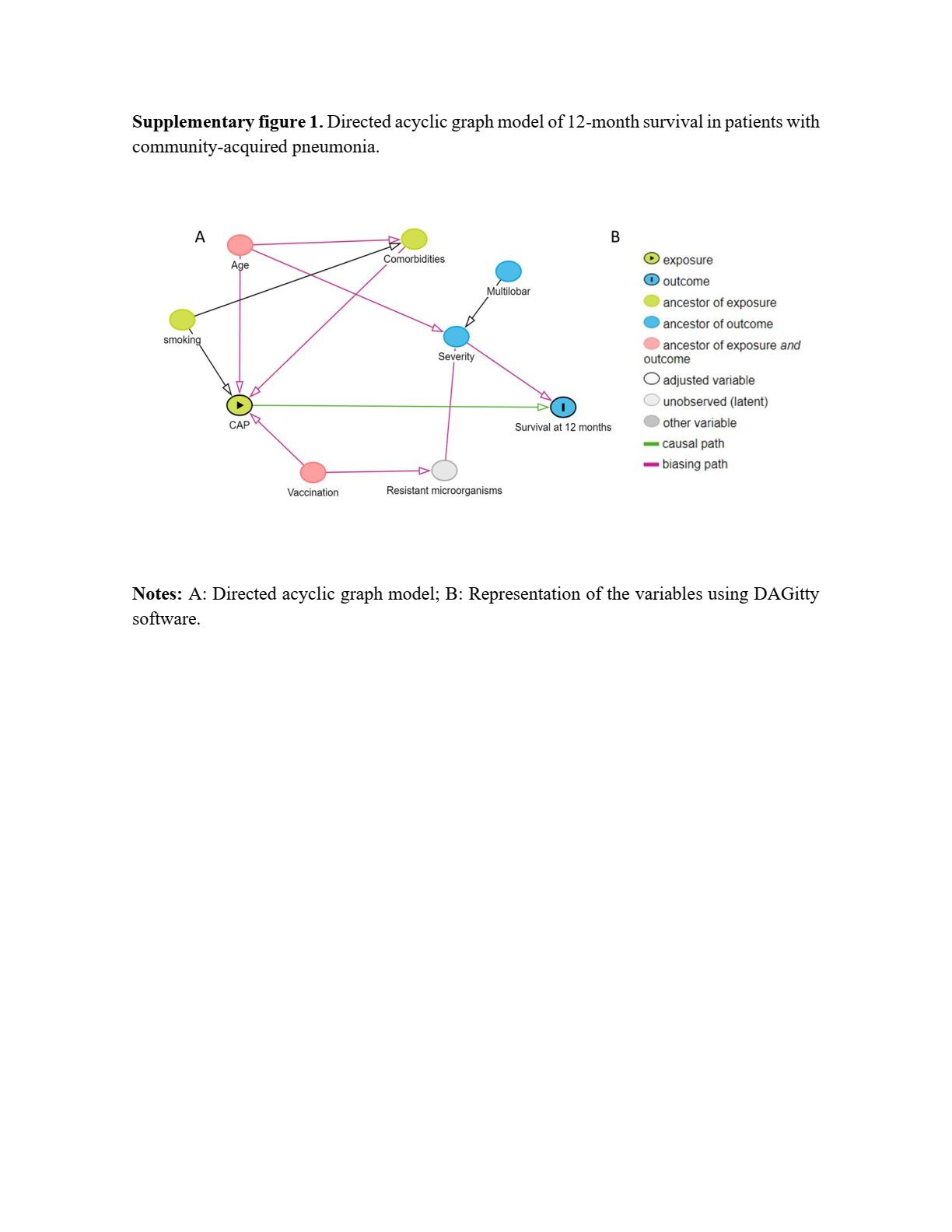


Supplementary Figure 2 Kaplan-Meier survival curves of hospitalized adult patients with community-acquired pneumonia according to sex. The p-value represents the statistically significant difference between men and women in survival at 3, 6, and 12 months.


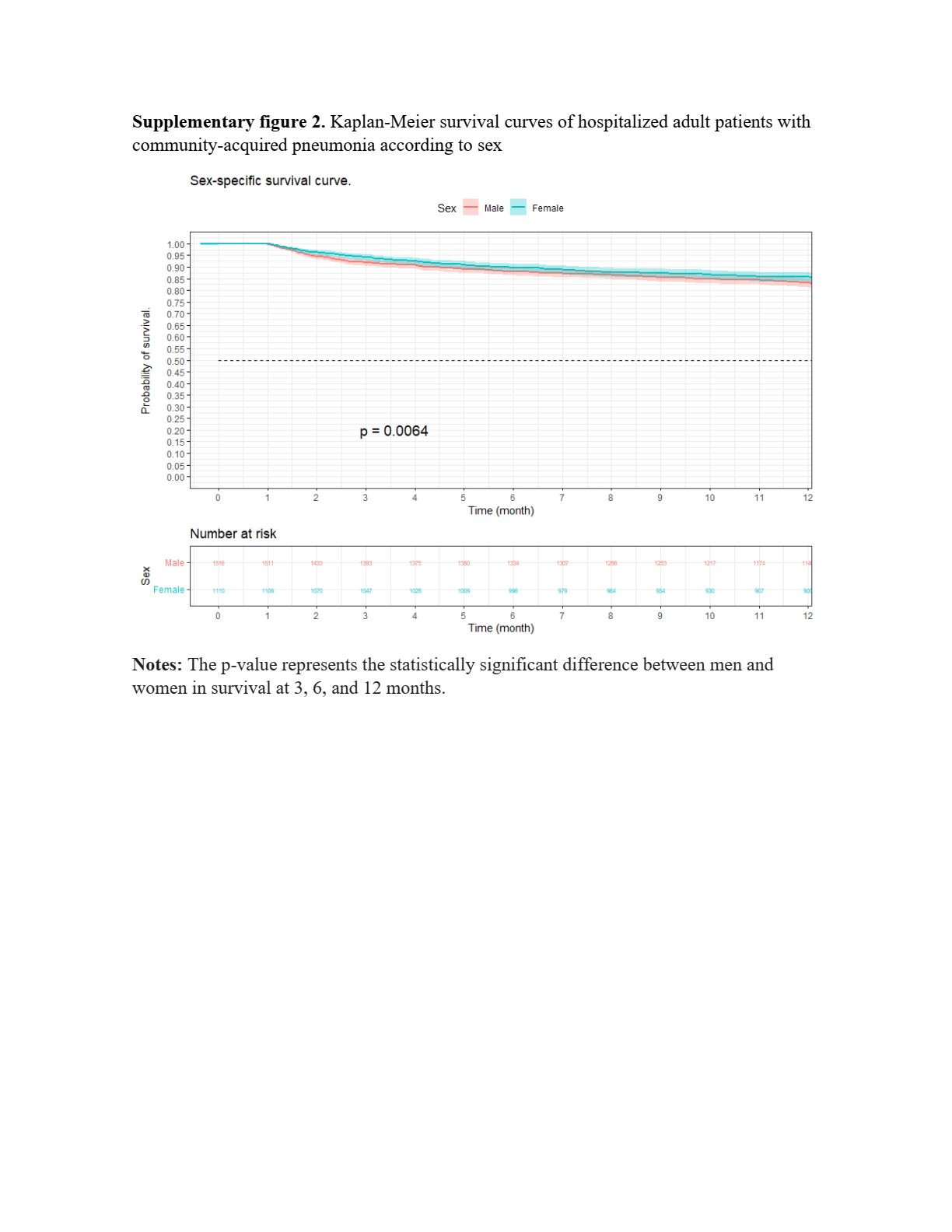


Supplementary Figure 3 Nelson-Aalen risk function estimation curve in hospitalized patients with community-acquired pneumonia according to comorbidity.


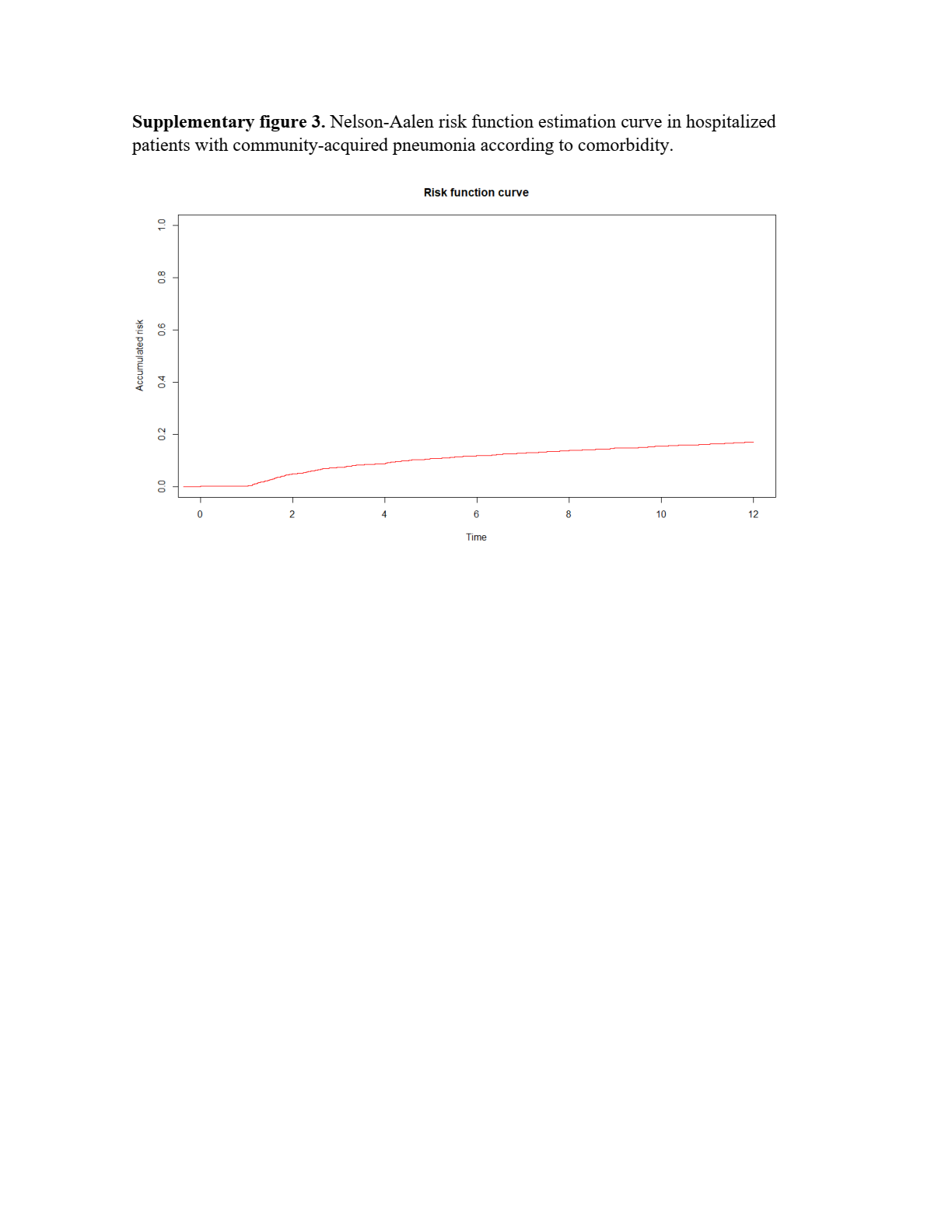


Supplementary Figure 4 Kaplan-Meier survival curves of patients > 65 years of age hospitalized with community-acquired pneumonia according to comorbidity.


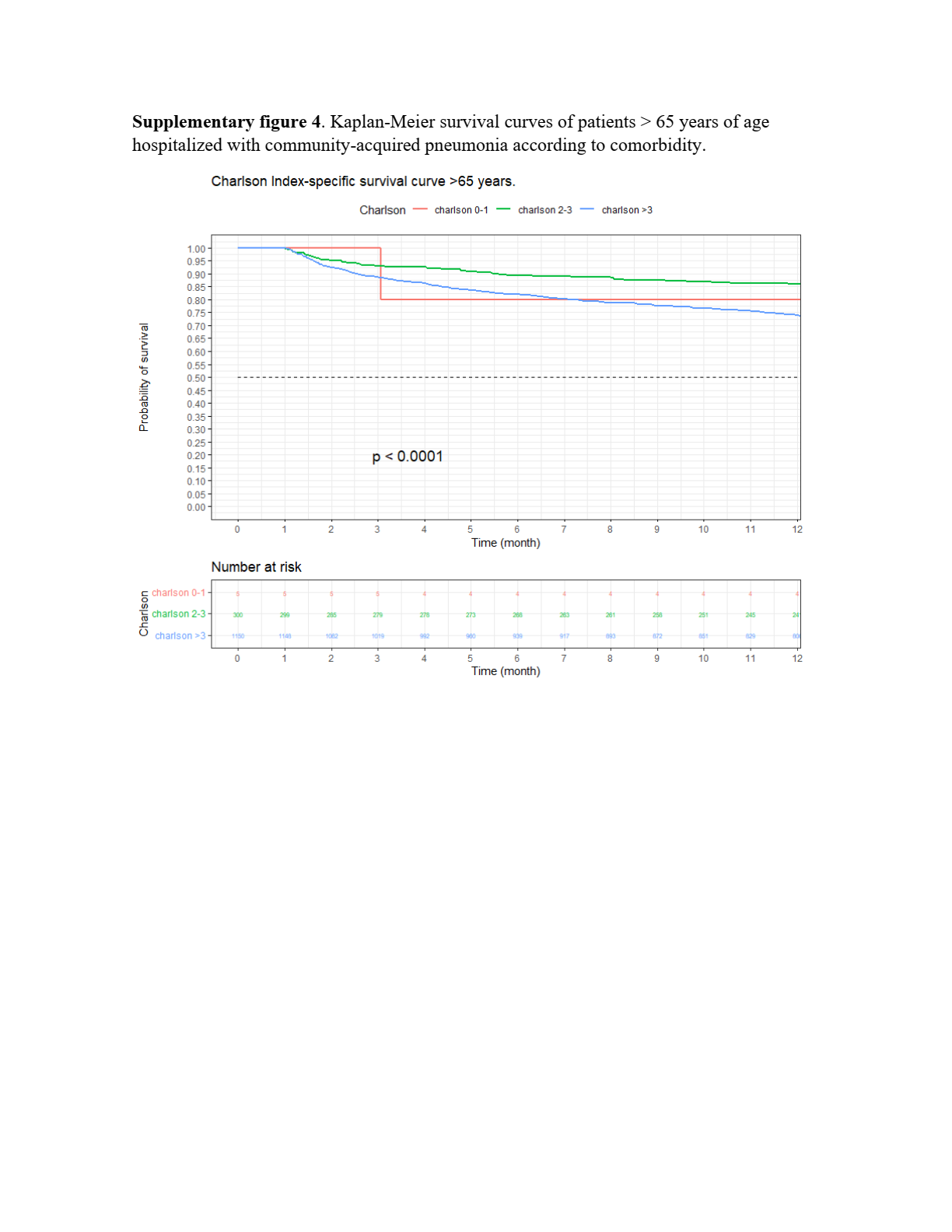


Supplementary Figure 5 Kaplan-Meier survival curves of patients < 65 years of age hospitalized with community-acquired pneumonia according to comorbidity.


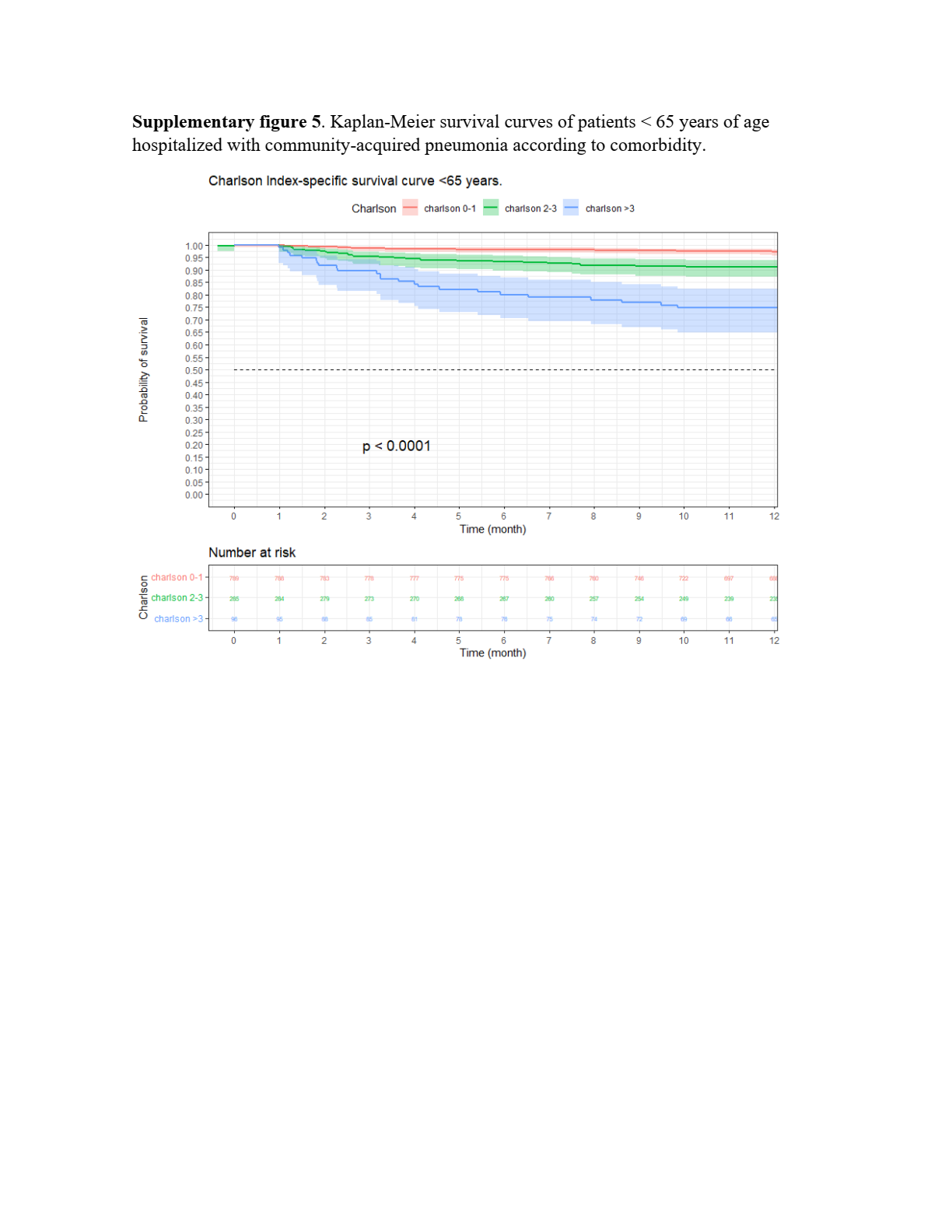

Supplement: Supplementary file 1 [file mmc1.docx]
